# Cuproptosis and its potential role in musculoskeletal disease

**DOI:** 10.3389/fcell.2025.1570131

**Published:** 2025-04-11

**Authors:** Ziyang Xiang, Huiling Mei, Honglin Wang, Xiaoyue Yao, Ji Rao, Wentao Zhang, Aoshuang Xu, Lin Lu

**Affiliations:** ^1^ Department of Orthopedics, Renmin Hospital of Wuhan University, Wuhan, China; ^2^ Department of Rheumatology and Immunology, Union Hospital, Tongji Medical College, Huazhong University of Science and Technology, Wuhan, China; ^3^ Department of Orthopaedics Surgery, Union Hospital, Tongji Medical College, Huazhong University of Science and Technology, Wuhan, China; ^4^ Institute of Hematology, Union Hospital, Tongji Medical College, Huazhong University of Science and Technology, Wuhan, China

**Keywords:** cuproptosis, osteoporosis, osteoarthritis, osteosarcoma, intervertebral disc degeneration, spinal cord injury, osteomyelitis

## Abstract

Cuproptosis, a recently identified form of copper-dependent cell death, arises from intracellular copper dyshomeostasis. As an essential trace element, copper plays a critical role in bioenergetic metabolism, redox regulation, and synaptic transmission. However, excessive copper exerts cytotoxic effects through multiple pathways, including increased reactive oxygen species (ROS) production, apoptotic cascade activation, necrotic membrane rupture, inflammatory responses, and mitochondrial dysfunction. Distinct from other cell death mechanisms, cuproptosis is characterized by copper ion binding to acetylated mitochondrial respiratory chain proteins, leading to pathogenic protein aggregation, iron-sulfur cluster depletion, and cellular collapse. Emerging evidence underscores aberrant copper accumulation and resultant proteotoxic stress as pivotal contributors to the pathogenesis of multiple musculoskeletal pathologies, including osteoporosis, osteoarthritis, sarcopenia, osteosarcoma, intervertebral disc degeneration, spinal cord injury, and biofilm-associated orthopedic infections. Understanding the spatiotemporal regulation of cuproptosis may provide novel opportunities for advancing diagnostic and therapeutic approaches in orthopedic medicine. This review synthesizes current insights into the molecular mechanisms of cuproptosis, its pathogenic role in musculoskeletal diseases, and the potential for biomarker-driven therapeutic interventions.

## 1 Introduction

Copper is an indispensable trace metal element in biological systems, extensively involved in critical physiological processes including energy metabolism, antioxidant defense, and connective tissue formation (Chen L. et al., 2022; Tian et al., 2023). As a cofactor for metalloenzymes such as cytochrome C oxidase (COX), superoxide dismutase (SOD), and lysyl oxidase (LOX), copper plays pivotal roles in maintaining mitochondrial respiratory chain function, scavenging free radicals, and promoting collagen cross-linking, which are essential for skeletal development, joint stability, and muscular contractility (Chen H. et al., 2024; Lutsenko et al., 2025). However, the strong redox activity of copper endows it with dose-dependent dual effects at the cellular level: under physiological concentrations, copper supports normal cellular functions through precise homeostatic regulation networks, whereas excessive accumulation triggers irreversible cellular damage via reactive oxygen species (ROS) generation and direct proteotoxicity (Tang et al., 2024; Tsvetkov et al., 2022; Wang J. et al., 2024; Chen J. et al., 2024). Chronic copper metabolism disorders have been well-documented in association with Wilson’s disease, neurodegenerative disorders, and various malignancies (Gromadzka et al., 2024; Gromadzka et al., 2020; Wang A. et al., 2023).

Recent advances in understanding metal-dependent apoptosis have revealed cuproptosis as a novel form of programmed cell death (Evans, 1973; Wu H. et al., 2024). In 2022, Tsvetkov et al. systematically characterized cuproptosis, revealing its core mechanism involving direct binding of excess copper ions to lipoylated proteins in the tricarboxylic acid (TCA) cycle, leading to mitochondrial protein aggregation and metabolic collapse (Tsvetkov et al., 2022). The initiation of cuproptosis strictly depends on spatiotemporal dynamics of intracellular copper concentrations (Doguer et al., 2018). Distinct from apoptosis, necroptosis, or ferroptosis, cuproptosis exhibits unique molecular signatures: caspase-independent execution, absence of lipid peroxide accumulation, coupled with mitochondrial swelling, profound dysregulation of lipoic acid metabolism pathways, and systemic failure of energy metabolism hubs (Tong et al., 2022; Tsvetkov et al., 2022) (Table 1). These features suggest cuproptosis may represent a specialized defense mechanism against metabolic imbalance, though its precise regulatory logic in tissue homeostasis and disease pathogenesis requires further elucidation.

**TABLE 1:** Similarities and differences among cuproptosis and other forms of cell death.

Feature	Cuproptosis	Pyroptosis	Necroptosis	Autophagy	Apoptosis	Ferroptosis
Definition	Copper-dependent cell death triggered by excess copper binding to lipoylated proteins, leading to mitochondrial dysfunction	Inflammatory cell death mediated by gasdermin pore formation and caspase-1 activation	Programmed necrosis mediated by RIPK1, RIPK3, and MLKL, leading to membrane rupture	Self-degradative process for recycling cellular components, which can lead to cell death if excessive	Programmed cell death characterized by caspase activation, DNA fragmentation, and cell shrinkage	Iron-dependent cell death driven by lipid peroxidation and oxidative stress
Trigger	Excess copper accumulation	Inflammasome activation (e.g., by pathogens or danger signals)	TNF, TLRs, viral infections, or other necroptotic stimuli	Nutrient deprivation, stress, or damaged organelles	Intrinsic (mitochondrial) or extrinsic (death receptor) apoptotic signals	Iron overload, GPX4 inhibition, or lipid peroxidation
Key Regulators	FDX1, lipoylated TCA cycle proteins	Caspase-1, gasdermin D (GSDMD)	RIPK1, RIPK3, MLKL	ATG proteins, LC3, Beclin-1	Caspases (e.g., caspase-3, -8, -9), Bcl-2 family	GPX4, ACSL4, iron metabolism
Mechanism	Copper binding to lipoylated proteins, mitochondrial dysfunction	Caspase-1 cleavage, pore formation	RIPK3-MLKL-mediated membrane rupture	Lysosomal degradation of cellular components	Caspase activation, DNA fragmentation	Lipid ROS accumulation, membrane damage
Biomarkers	Copper levels, lipoylated proteins, FDX1	Caspase-1 activation, GSDMD cleavage, IL-1β release	Phospho-MLKL, RIPK3 activation	LC3-II, SQSTM1/p62 degradation	Caspase activation, DNA fragmentation	Lipid ROS, GPX4 inhibition, iron accumulation
Functional Outcome	Cell death due to copper toxicity and metabolic disruption	Host defense against pathogens, but can cause tissue damage	Alternative cell death pathway when apoptosis is blocked	Cell survival under stress, but can lead to cell death if excessive	Controlled cell removal without inflammation	Cell death due to oxidative damage and lipid peroxidation

The musculoskeletal system, as the structural foundation of locomotor function, critically relies on functional coordination among osteoblasts, osteoclasts, chondrocytes, and myocytes (Luo et al., 2024). Clinical studies reveal elevated synovial fluid copper levels in osteoarthritis patients, positively correlated with articular cartilage degradation (Yazar et al., 2005), while osteoporosis patients exhibit serum copper fluctuations closely linked to bone density dynamics (Wei et al., 2022). Additionally, muscle degenerative disease models demonstrate aberrant subcellular localization of copper transporters (Jackson, 2009). While traditional paradigms attribute these phenomena to copper-mediated oxidative damage or inflammatory cascades, emerging evidence implicates cuproptosis in disease pathogenesis (Li C. et al., 2023; Tsvetkov et al., 2022). Experimental studies demonstrate that the copper ionophore elesclomol specifically induces osteoblastic cuproptosis through mitochondrial lipoylated protein aggregation and impaired bone matrix mineralization (Muthurangan et al., 2023). Conversely, copper chelators effectively suppress synovial cell death and inflammatory cytokine release in rheumatoid arthritis models (Zhao et al., 2022). These findings collectively point to an underappreciated pathological axis: copper homeostasis imbalance may drive musculoskeletal disorders through cell type-specific cuproptosis activation.

To clarify the roles of copper homeostasis and cuproptosis in musculoskeletal disorders, this review comprehensively outlines copper metabolism and regulatory mechanisms, examines the molecular basis of cuproptosis, and systematically analyzes its research progress across these diseases. These findings aim to provide novel insights for developing precision therapeutic strategies targeting musculoskeletal pathologies.

## 2 Copper and cuproptosis

### 2.1 Physiological roles of copper

Copper, an essential trace element in humans, plays critical roles in numerous biological processes including reactive oxygen species (ROS) detoxification, cellular energy metabolism, iron absorption, and signal transduction (Tang et al., 2024). It is obtained primarily from dietary sources such as shellfish, meat, seeds, nuts, lentils, and leafy greens (Burkhead et al., 2009). As an indispensable micronutrient for maintaining cellular homeostasis, copper is involved in cell proliferation, angiogenesis, and metastasis, necessitating strict regulatory mechanisms to prevent toxicity (Han et al., 2024; Tsvetkov et al., 2022). The recommended daily copper intake for adults ranges from 0.8 to 2.4 mg to maintain systemic copper homeostasis (Chen W. et al., 2022). Maintaining a dynamic equilibrium of copper levels is essential for normal cellular function and metabolism (Locatelli and Farina, 2025), while disruptions in copper homeostasis can lead to severe pathological conditions (An et al., 2022; Burkhead et al., 2009).

Copper deficiency impairs the activity and functionality of cuproenzymes, resulting in anemia, increased vascular and skeletal fragility, cerebral atrophy, impaired endocrine function, and neurological deficits. Additionally, it has been linked to depigmentation disorders such as vitiligo. Conversely, copper overload is cytotoxic. Chronic excess intake interferes with iron and zinc absorption, potentially leading to deficiencies in these elements. In severe cases, copper toxicity can result in hepatic injury and systemic dysfunction (Han et al., 2024).

### 2.2 Systemic copper metabolism

Mammalian copper homeostasis is maintained through a tightly regulated network of proteins, including ceruloplasmin in plasma, copper transporter 1 (CTR1), the cytosolic copper chaperone ATOX1, copper efflux proteins ATP7A/B, and mitochondrial copper chaperones such as CCS, SCO1, SCO2, COX11, and COX17. Together, these components orchestrate copper absorption, transport, storage, and excretion (Tsvetkov et al., 2022). Dietary copper absorption primarily occurs in the small intestine, a process dependent on CTR1 located on the apical membrane of intestinal epithelial cells (Kardos et al., 2018). The six-transmembrane epithelial antigen of the prostate (STEAP) and duodenal cytochrome B (Dcytb) are involved in reducing divalent copper ions (Cu^2+^) to monovalent copper ions (Cu^+^) for transport via CTR1 (Teschke and Eickhoff, 2024; Wapnir, 1998), while copper efflux across the basolateral membrane requires ATP7A. As a critical copper transporter, CTR1 expression is regulated by copper levels: its expression is suppressed under high copper conditions and enhanced under low copper conditions, indicating a negative feedback mechanism governing intracellular copper absorption and utilization (Tsvetkov et al., 2022; Zhu et al., 2024).

**FIGURE 1:**
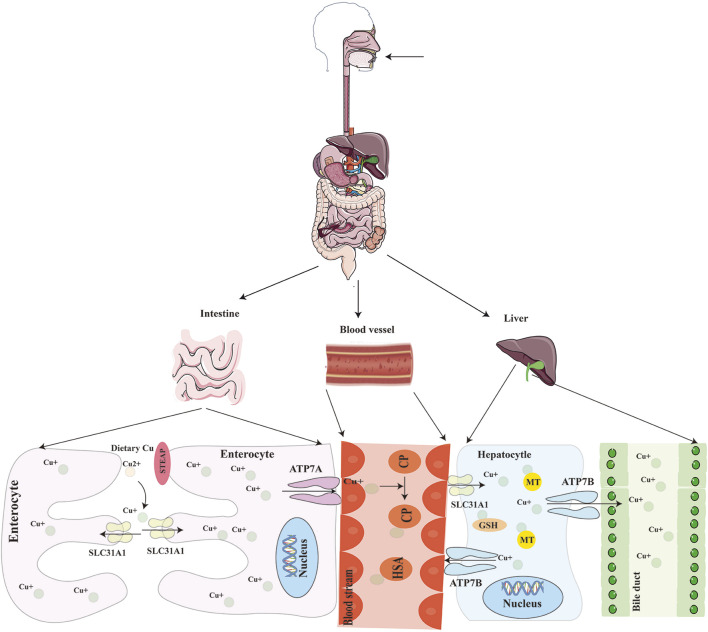
Schematic of systemic copper metabolism. The body absorbs copper mostly through the small intestine, where it is then transported by blood to the liver for excretion into the bile. STEAP: six-transmembrane epithelial antigen of the prostate; SLC31A1(CTR1): copper transporter 1; ATP7A/B: ATPase copper transporting alpha/beta.

Once absorbed, copper is released into the portal circulation via ATP7A and bound to metalloproteins such as albumin (Qiu et al., 2024). When copper is transported to the liver, hepatocytes uptake the majority of copper via CTR1 and store it intracellularly with the involvement of metallothioneins (MT1, MT2). Excess copper is secreted into bile via the ATOX1/ATP7B/ceruloplasmin pathway in vesicular form, ultimately excreted through bile secretion into the digestive tract—a major route for endogenous copper elimination (Tsvetkov et al., 2022) (Figure 1). If peripheral copper levels are insufficient to maintain homeostasis, ATP7B exports stored hepatic copper into systemic circulation, where it is bound to ceruloplasmin for transport to specific tissues or organs for utilization (An et al., 2022; Qiu et al., 2024). Additionally, low-molecular-weight copper ligands such as aspartate, histidine, and cysteine are implicated in peripheral copper absorption and utilization (Viktorinova, 2017).

In peripheral tissues, copper ions are either sequestered by compounds like metallothioneins or targeted for utilization by chaperones such as ATOX1, COX17, and CCS (Liu et al., 2023). Copper homeostasis is maintained through a dynamic balance between absorption and excretion—high copper intake triggers reduced absorption and increased excretion, whereas low intake leads to enhanced absorption and reduced excretion. Furthermore, copper serves as a cofactor for key metabolic enzymes involved in critical physiological processes, including mitochondrial respiration, redox reactions, antioxidant metabolism, and the absorption and utilization of other biomolecules. These enzymes play indispensable roles in cellular respiration, endocrine function, and the development and maintenance of the central nervous system (Fukai et al., 2018; Leuci et al., 2025; Tang et al., 2024).

### 2.3 Cellular copper metabolism

Cellular copper homeostasis is the foundation for macroregulatory control of copper homeostasis. Dysregulation of copper balance disrupts the intracellular environment, leading to cellular damage and death. In multicellular organisms, copper metabolism operates at both systemic and cellular levels. Copper acts as an electron acceptor or donor, participating extensively in diverse biochemical reactions. The liver, which contains the highest copper concentration in humans, serves as the primary regulatory organ for copper metabolism (Wang A. et al., 2023; Zhou et al., 2023). To prevent copper toxicity and maintain intracellular equilibrium, copper levels are tightly controlled by a sophisticated network of copper-dependent proteins, including cuproenzymes, copper chaperones, and membrane transporters.

Copper transporter 1 (CTR1) and STEAP collaboratively mediate copper uptake into cells, while copper chaperones—such as the copper chaperone for superoxide dismutase (CCS), antioxidant-1 (ATOX1), and the copper chaperone for cytochrome c oxidase (COX17)—orchestrate intracellular copper trafficking (Festa and Thiele, 2011; Gromadzka et al., 2020; Zhang et al., 2024). CCS delivers copper to superoxide dismutase 1 (SOD1), which scavenges reactive oxygen species (ROS) and maintains redox balance (Zhang et al., 2024). ATOX1 binds Cu^+^ and transfers it to ATP7A and ATP7B in the trans-Golgi network, facilitating cuproenzyme biosynthesis (Wu J. et al., 2023). Under conditions of copper excess, ATP7A and ATP7B export surplus copper to the plasma membrane (Feng et al., 2024). COX17 targets Cu^+^ to the mitochondrial intermembrane space, where it is incorporated into cytochrome c oxidase (CCO) via SCO1, supporting cellular respiration (Roy and Lutsenko, 2024).

**FIGURE 2:**
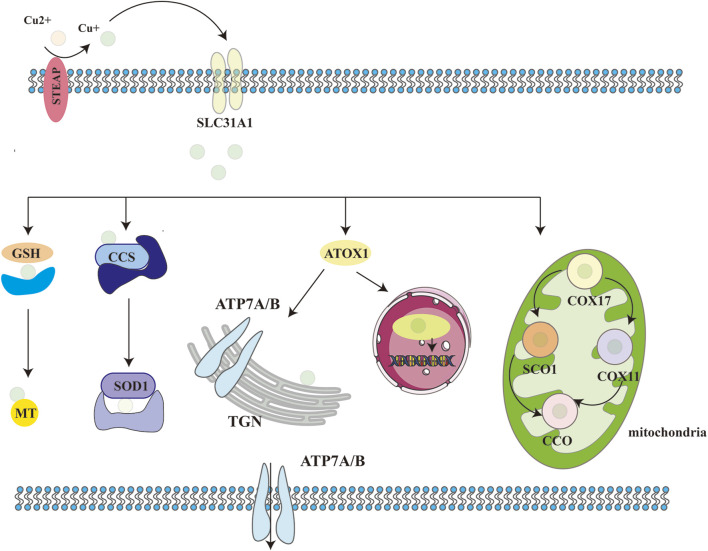
Schematic of cellular copper metabolism. Biological processes in which copper ions are involved in cells. STEAP: six-transmembrane epithelial antigen of the prostate; CTR1:copper transporter 1; CCS: copper chaperone for superoxide dismutase; SOD1: superoxide dismutase 1; COX17: cytochrome C oxidase copper chaperone 17; COX11: cytochrome C oxidase copper chaperone 11; CCO: cytochrome C oxidase; SCO1/2: synthesis of cytochrome C oxidase 1/2; ATOX1: antioxidant 1; ATP7A/B: ATPase copper transporting alpha/beta; TGN:trans-Golgi network.

Additionally, copper-binding molecules such as glutathione and metallothionein 1/2 (MT1/MT2) neutralize excess copper ions, ensuring cellular stability (Jia et al., 2024). When needed, Cu^+^ is oxidized to Cu^2+^by ROS. Due to its low affinity for MT, Cu^2+^ is released into the cytosol to participate in metabolic regulation. Intracellular Cu^+^ is exported via ATP7A through endosomal/Golgi pathways or directly expelled by ATP7B. During copper overload, ATP7B facilitates copper sequestration in hepatocytes, directing it toward biliary excretion to maintain systemic homeostasis. This intricate interplay among cuproenzymes, chaperones, and transporters ensures that cellular copper levels remain within physiological ranges, preventing both deficiency and toxicity (Figure 2).

## 3 Mechanism of cuprotosis

The discovery of copper-induced cell death dates back to the early 1980s (Halliwell and Gutteridge, 1984). Studies indicate that elevated copper levels promote reactive oxygen species (ROS) generation, triggering oxidative stress and DNA damage, ultimately leading to cell death (Gaetke and Chow, 2003). These findings spurred further investigation into the molecular mechanisms of copper-induced cell death and its implications for human health. Conflicting evidence suggests that, beyond ROS accumulation, excess copper may also induce cell death via apoptosis or caspase-independent pathways (Nagai et al., 2012; Tardito et al., 2011).

In March 2022, Tsvetkov et al. (2022) published groundbreaking research elucidating a novel mechanism of copper-induced cell death, termed “cuprotosis”. This distinct form of cell death is driven by excessive copper ions. The authors demonstrated that treatment with the copper ionophore elesclomol (ES) induced cell death, which could only be rescued by copper chelators, whereas inhibitors targeting apoptosis, necroptosis, oxidative stress, ROS-induced cell death, or ferroptosis showed no protective effect. These findings confirm that cuprotosis operates through unique mechanisms and signaling pathways distinct from other known cell death modalities. Crucially, cuprotosis is regulated by mitochondrial respiration. Studies reveal that cells reliant on mitochondrial respiration exhibit nearly 1,000-fold higher sensitivity to copper ionophores compared to glycolysis-dependent cells (Tsvetkov et al., 2022), underscoring the pivotal role of mitochondrial respiration in cuprotosis. This highlights a strong link between cuprotosis and the tricarboxylic acid (TCA) cycle. During cuprotosis, intracellular copper binds to lipoylated components of the TCA cycle, inducing aggregation of copper-bound lipoylated mitochondrial proteins. This disrupts the TCA cycle, impairing cellular energy production. The aggregation of these proteins, coupled with subsequent depletion of Fe-S cluster proteins—essential cofactors for electron transport and enzymatic reactions (Lill and Freibert, 2020)—triggers proteotoxic stress and culminates in cell death (Figure 2).

**FIGURE 3:**
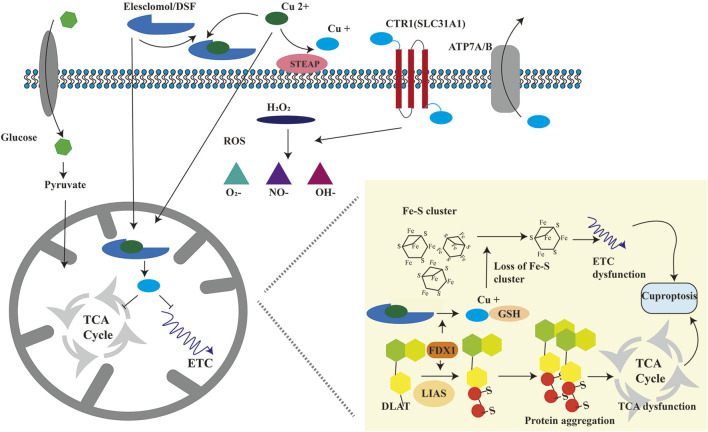
Schematic diagram of the molecular mechanism of cuproptosis. Cu^+^ is transported into the cell via SLC31A1 and out of the cell via ATP7A/B. Cu^2+^ is transported into the cell by copper ionophores, including ES and DSF. Cu^2+^ is reduced to Cu^+^ by FDX1. On the one hand, excessive Cu^+^ binds to fatty acid acylated mitochondrial proteins, causing the oligomerization of fatty acid acylated proteins, leading to proteotoxic stress, blocking TCA (tricarboxylic acid) cycle, and causing cuproptosis. On the other hand, excessive Cu^+^ can reduce iron-sulfur cluster proteins, resulting in abnormal electron transport chain and causing cuproptosis. FDX1: ferredoxin 1; DLAT: dihydrolipoic acid transacetylase; LIAS: Lipoic acid synthetase.

To elucidate how ES-induced cuprotosis targets the TCA cycle, Tsvetkov et al. conducted a genome-wide CRISPR-Cas9 loss-of-function screen, identifying ten cuprotosis-related genes. These include seven positive regulators: ferredoxin 1 (FDX1), dihydrolipoamide S-acetyltransferase (DLAT), lipoic acid synthase (LIAS), dihydrolipoamide dehydrogenase (DLD), lipoyltransferase 1 (LIPT1), pyruvate dehydrogenase E1 subunit alpha 1 (PDHA1), and pyruvate dehydrogenase E1 subunit beta (PDHB); and three negative regulators: metal regulatory transcription factor 1 (MTF1), glutaminase (GLS), and cyclin-dependent kinase inhibitor 2A (CDKN2A) (Zhou et al., 2024) (Table 2). FDX1 and protein lipoylation are central to ES-induced cuprotosis (Scheiber et al., 2014). FDX1 encodes a reductase that reduces Cu^2+^ to the more toxic Cu^+^. Protein lipoylation, a highly conserved lysine post-translational modification, regulates protein function by attaching a lipoic acid moiety to lysine residues. This modification occurs exclusively in four multi-enzyme complexes, such as the pyruvate dehydrogenase (PDH) complex (of which DLAT is a component) (Magistrato et al., 2019). Tsvetkov et al. (2022) further demonstrated that FDX1 expression correlates strongly with lipoylated proteins (Lip-DLAT) and that FDX1 knockout abolishes protein lipoylation, identifying FDX1 as an upstream regulator of this process (Wang W. et al., 2023; Zhou, et al., 2023). Subsequent experiments revealed that Cu^+^directly binds to Lip-DLAT, inducing its oligomerization. Concurrently, ES-treated cells exhibited reduced Fe-S cluster protein levels. The researchers also found that CuCl_2_ mimics ES by inducing cuprotosis, depleting glutathione (GSH), and upregulating CTR1 (SLC31A1), thereby potentiating CuCl-induced cell death (Figure 3) (Wang J. et al., 2024). In summary, cuprotosis is driven by excessive intracellular copper binding to lipoylated TCA cycle proteins, leading to oligomerization, Fe-S cluster protein loss, proteotoxic stress, and eventual cell death.

**TABLE 2:** Regulating factors of Cuproptosis.

Gene	Full name	Subcellular localization	Functional description
FDX1	Ferredoxin 1	Mitochondrial matrix	Core regulatory gene of cuproptosis; reduces Cu^2+^ to toxic Cu^+^, directly triggering mitochondrial metabolic collapse and cell death
LIPT1	Lipoyltransferase 1	Mitochondrial matrix	Mediates lipoic acid transfer; copper overload disrupts its function, leading to inactivation of mitochondrial enzyme complexes (e.g., PDC)
DLAT	Dihydrolipoamide S-Acetyltransferase	Mitochondrial matrix (PDC component)	Copper binding induces DLAT aggregation, disrupting pyruvate dehydrogenase complex (PDC) function and energy metabolism
LIAS	Lipoic Acid Synthase	Mitochondrial matrix	Catalyzes lipoic acid synthesis; copper inhibits LIAS activity, impairing mitochondrial oxidative phosphorylation
DLD	Dihydrolipoamide Dehydrogenase	Mitochondrial matrix (α-KGDH complex)	Copper-induced DLD dysfunction disrupts α-ketoacid dehydrogenase complexes and causes ROS accumulation
PDHA1	Pyruvate Dehydrogenase E1α	Mitochondrial matrix (PDC component)	Copper binding to PDHA1 disrupts PDC function, blocking the TCA cycle
PDHB	Pyruvate Dehydrogenase E1β	Mitochondrial matrix (PDC component)	Copper induces PDHB oligomerization, halting mitochondrial metabolism and promoting cell death
MTF1	Metal Regulatory Transcription Factor 1	Nucleus	Regulates metal homeostasis genes (e.g., metallothioneins); copper overload may activate stress-induced death via MTF1 signaling
GLS	Glutaminase	Mitochondria/Cytoplasm	Copper inhibits GLS activity, blocking glutamine metabolism and exacerbating mitochondrial stress
CDKN2A	Cyclin-Dependent Kinase Inhibitor 2A	Nucleus	Modulates cuproptosis sensitivity indirectly by regulating cell cycle or stress response pathways
SLC31A1	Solute Carrier Family 31 Member 1	Cell membrane	Encodes copper transporter CTR1, regulating copper uptake and cuproptosis thresholds
ATP7A/B	ATPase Copper Transporting A/B	Golgi apparatus/Cell membrane	Copper-transporting ATPases responsible for efflux/compartmentalization; dysfunction leads to copper accumulation and death signaling

## 4 Key signaling pathways and mediators in cuprotosis

Cuprotosis involves multiple signaling pathways and interactions with critical proteins. Copper induces mitochondrial dysfunction, activating intrinsic apoptotic pathways. Key mitochondrial proteins, including cytochrome C, apoptosis-inducing factor (AIF), and Bcl-2 family members, have been implicated in copper-induced cell death (Nam et al., 2018). Copper ions also activate stress- and apoptosis-related pathways such as MAPK (mitogen-activated protein kinase), p38 MAPK, and JNK (c-Jun N-terminal kinase) (Wittung-Stafshede, 2022). Studies (Hatori and Lutsenko, 2016; Valko et al., 2005) suggest that copper enhances ROS production, which subsequently triggers cell death via NF-κB and p53 pathways. Fogh et al. (2014) further demonstrated that protein kinase C (PKC) modulates cytoskeletal dynamics and cell adhesion during copper-induced cellular alterations.

## 5 Cuproptosis in musculoskeletal diseases

### 5.1 Osteoarthritis and cuproptosis

Osteoarthritis (OA), a prevalent chronic degenerative joint disease, imposes significant physical and economic burdens on aging populations. Its pathological mechanisms involve chronic inflammation predominantly affecting the synovium, articular cartilage, and subchondral bone, leading to cartilage degradation, subchondral sclerosis, and osteophyte formation (Chen et al., 2017). OA most commonly affects the knees and hips, with risk factors including age, obesity, and severe joint injury (Li F. et al., 2022). Clinically, OA management remains challenging, with joint replacement serving as the primary intervention for end-stage disease. However, increasing rates of arthroplasty, coupled with complications such as prosthesis failure, infection, and periprosthetic fractures, underscore the urgent need for early therapeutic strategies to delay disease progression (Hunter et al., 2020).

Copper metabolism exhibits a complex relationship with OA pathogenesis (Yazar et al., 2005). Under physiological conditions, copper ions act as essential cofactors for enzymes critical to cartilage homeostasis, including lysyl oxidase (LOX), superoxide dismutase (SOD), cytochrome C oxidase (Cox) (Yang J. et al., 2023). Moderate Cu^2+^ levels promote cartilage regeneration and bone-cartilage interface repair, mitigating tissue damage (Zhang et al., 2024). Conversely, excessive copper induces ROS generation, lipid peroxidation, and inflammatory cascades, exacerbating cartilage and bone degradation. Cuproptosis—copper-dependent cell death—is driven by intracellular copper accumulation, where Cu^+^ binds to lipoylated components of the tricarboxylic acid (TCA) cycle, causing protein aggregation, TCA cycle disruption, proteotoxic stress, and ultimately cell death (Zhou et al., 2021). Notably, copper accumulation has been observed in severely degraded cartilage, correlating with accelerated joint damage (Guan et al., 2023).

Current research on cuproptosis in OA focuses on copper-related genes (CRGs) as potential biomarkers and therapeutic targets (Wang W. et al., 2023). A predictive model for OA risk and progression has been established using four CRGs (DBT, DLST, FDX1, LIPT1), where FDX1 and LIPT1 are upregulated in OA, while DBT and DLST are elevated in healthy controls (Nong et al., 2023). CRGs influence OA through multiple pathways (Guan et al., 2024; He et al., 2024; Yu et al., 2024): (a). Mitochondrial Dysfunction: Impaired mitochondrial activity in joint cells (e.g., chondrocytes, synoviocytes) during OA exacerbates inflammation, apoptosis, and metabolic dysregulation. CRGs, closely linked to mitochondrial function, may amplify these pathologies when dysregulated. (b). Copper Homeostasis Disruption: CRGs modulate intracellular copper balance, impacting chondrocyte survival and mitochondrial integrity. (c). Immune Regulation: CRGs correlate with immune cell infiltration and inflammatory responses in OA synovium, suggesting roles in disease progression. (d). Protein Aggregation: CRGs regulate the expression of mitochondrial proteins (e.g., lipoylated, copper-sensitive proteins), influencing chondrocyte susceptibility to cuproptosis. (e). Metabolic Pathway Interference: CRG-associated pathways, such as the TCA cycle, are disrupted in OA, leading to metabolite accumulation and chondrocyte dysfunction.

In addition, synovitis, a hallmark of OA, drives cartilage destruction. Five CRGs (FDX1, LIPT1, PDHA1, PDHB, CDKN2A) are markedly upregulated in OA synovial tissues (Nong et al., 2023). Single-cell RNA sequencing reveals dynamic shifts in synovial cell populations during OA progression, with these CRGs emerging as candidate biomarkers or therapeutic targets for synovitis (Chang et al., 2023).

For therapeutic strategies, nanoparticle-based approaches show promise in cartilage tissue engineering. Li et al. (2024) constructed copper-based bioactive nanoparticles with Cuprorivaite as the carrier and demonstrated its potential in protecting cartilage in OA progression by inhibiting inflammation, oxidative stress, and copper-induced cell death. Zhu et al. (2022) developed a multifunctional thermoresponsive gel (HPP@Cu gel), which efficiently scavenged free radicals through copper nanodots (Cu NDs) and induced polarization of macrophages from M1 to M2 phenotypes at the inflammatory site, significantly reducing OA-induced cartilage degeneration and production of inflammatory factors. Additionally, curcumin, a natural compound, has been utilized as a copper shuttle protein to kill cancer cells intracellularly via copper transport mechanisms (Zhang et al., 2016). However, researchers have focused more on copper’s catalytic roles in various applications, with fewer studies investigating the therapeutic effects at different copper levels. Therefore, this field still requires further exploration and development.

### 5.2 Osteoporosis and cuproptosis

Osteoporosis (OP) is a systemic skeletal disorder characterized by reduced bone mass and microarchitectural deterioration, leading to increased bone fragility and fracture susceptibility (Lane et al., 2000). Its pathogenesis is multifactorial, involving complex interactions of hormonal, cellular, and molecular mechanisms (Wang et al., 2009). Bone homeostasis in healthy adults is maintained by a dynamic balance between osteoblast-mediated bone formation and osteoclast-mediated bone resorption. In OP, this equilibrium is disrupted, often favoring excessive bone resorption over formation, resulting in progressive bone loss (Clynes et al., 2020). Key regulators of bone metabolism include hormones such as parathyroid hormone, estrogen, testosterone, and vitamin D, as well as cytokines like interleukins (ILs), tumor necrosis factors (TNFs), and osteoprotegerin (OPG) (Fischer and Haffner-Luntzer, 2022; Liu B. et al., 2022). Additionally, excessive reactive oxygen species (ROS) production damages bone cells via DNA lesions, protein oxidation, and cellular dysfunction (Iantomasi et al., 2023). Moreover, genetic predisposition, particularly polymorphisms affecting bone mineral density (BMD), and environmental factors such as nutritional deficiencies (e.g., calcium, vitamin D) further contribute to OP risk (Wang et al., 2009).

Emerging evidence demonstrates that the physiological hypoxic niche within bone confers cytoprotective effects against ROS-mediated oxidative damage, with the osseous tissue and medullary cavity inherently maintaining a state of natural hypoxia (Riegger et al., 2023). Both osteoblasts and osteoclasts—central orchestrators of skeletal homeostasis—possess intrinsic oxygen-sensing capacity through HIF-1ɑ signaling (Chen W. et al., 2022). This oxygen-sensitive pathway dichotomously regulates bone remodeling dynamics by coordinating anabolic/catabolic balance and modulating bone turnover rates (Knowles, 2015). However, chronic hypoxic stimulation paradoxically upregulates nuclear factor-κB ligand (RANKL) expression via HIF-1α/NF-κB transcriptional synergy, thereby disrupting homeostatic control over osteoclast differentiation (Zhang C. et al., 2023). Osteoporosis pathogenesis fundamentally represents a pathological cascade initiated by marrow microenvironmental destabilization and dysregulated intercellular crosstalk.

Cuproptosis may also alter the bone marrow microenvironment, impairing the osteogenic differentiation and promoting adipogenic differentiation of bone marrow mesenchymal stem cells (BMSCs) (Li D. et al., 2023). Mechanistically, cuproptosis-associated mitochondrial metabolic disturbances—particularly FDX1-mediated proteotoxic stress from impaired copper ion buffering—may drive disease progression through aberrant activation of apoptosis-executing pathways (Chen H. et al., 2024). Furthermore, reactive oxygen species (ROS) generated during cuproptosis intensify intracellular oxidative stress, directly damaging osteocytes while simultaneously triggering inflammatory responses that recruit immune cells to bone tissue, exacerbating bone destruction (Liu et al., 2024). This imbalance could reduce bone formation and increase fat deposition, ultimately lowering bone mineral density. Moreover, cuproptosis may further disrupt the expression of genes and proteins critical to bone metabolism, such as the osteoblast-specific transcription factors Runx2 and Osterix, as well as the RANKL/OPG system—a key molecular regulator of bone formation and resorption (Wang et al., 2024b). Additionally, cuproptosis may modify the composition of miRNAs within extracellular vesicles (EVs), which are internalized by neighboring bone cells, subsequently influencing their function (Sun et al., 2024). For instance, specific miRNAs may suppress osteoblast differentiation or enhance osteoclast activity (Li Q. C. et al., 2022). These mechanisms collectively contribute to osteoporosis pathogenesis.

Notably, certain osteoporosis therapeutics, such as bisphosphonates, may exert their effects partially through modulation of copper metabolism and cuproptosis pathways (Kanumakala et al., 2002). Building on the interplay between cuproptosis and osteoporosis, researchers are exploring novel therapeutic strategies to harness cuproptosis mechanisms for OP treatment. These include regulating copper metabolism, targeting cuproptosis-related genes and utilizing EVs to deliver miRNAs (Sun et al., 2024). The overarching goal is to fine-tune cuproptosis to preserve bone cell health and function, thereby advancing both therapeutic and preventive approaches for osteoporosis.

### 5.3 Sarcopenia and cuproptosis

The development of sarcopenia, characterized by age-related loss of skeletal muscle mass and strength, has been demonstrated to result from mitochondrial dysfunction, elevated reactive oxygen species (ROS) production, and denervation (Cruz-Jentoft et al., 2019). Skeletal muscle, a highly copper-dependent and metabolically active tissue, serves as a major reservoir for copper ions (Lutsenko, 2021). Copper is indispensable not only for the initial formation of skeletal muscle—particularly myoblast proliferation and differentiation—but also for post-differentiation copper redistribution within myocytes, mitochondrial copper trafficking, and the metabolic maintenance of myofibers (Cobine et al., 2021; McCann et al., 2022). As a copper-demanding organ, skeletal muscle exhibits abundant and dynamic copper metabolism. The widespread expression of CTR1 and ATP7A/B in skeletal muscle cells indicates that intracellular copper homeostasis adheres to universal physiological principles of copper regulation, governed by the balance between copper uptake and efflux (Jin et al., 2021).

Current studies reveal that copper overload may activate multiple cell death pathways, including apoptosis, pyroptosis, ferroptosis, and cuproptosis, while promoting α-synuclein aggregation (Li et al., 2019; Yuan and Larsson, 2023). These processes lead to direct degradation of myocellular components, degeneration of the neuromuscular junction (NMJ), and subsequent skeletal muscle atrophy. These metabolic programs involve distinct regulatory mechanisms: copper overload-induced myocyte apoptosis is primarily mediated by ROS, p53, and mitochondrial dynamin-related protein dysregulation (Chen et al., 2021; Nishikawa et al., 2021). Additionally, ROS facilitate copper overload-triggered pyroptosis by upregulating tumor necrosis factor-α (TNF-α) expression and activating caspases, a process potentially requiring copper-induced inflammatory cooperation (Jin et al., 2022; Wu Z. et al., 2023).

To date, four cuproptosis-related genes—PDHA1, DLAT, PDHB, and NDUFC1—have been identified as diagnostic biomarkers for age-related skeletal muscle atrophy (Zhu et al., 2023). Further research demonstrates that PDHB and NDUFC1 act as novel molecular targets to counteract myofiber senescence. Metformin hydrochloride, a therapeutic agent for muscle atrophy, requires NDUFC1 mediation for its regulatory effects (Jiang J. et al., 2023; Zhu et al., 2023). Moreover, immunologically relevant cuproptosis-associated genes are closely linked to musculoskeletal aging progression and may serve as technical options for early diagnosis of muscle atrophy, underscoring the potential role of copper overload-induced cuproptosis in skeletal muscle senescence (Lin et al., 2022). However, the precise mechanisms by which copper overload triggers cuproptosis in aged skeletal muscle fibers remain elusive, warranting further investigation.

### 5.4 Intervertebral disc degeneration and cuproptosis

Intervertebral Disc Degeneration (IVDD) is a predominant contributor to low back pain, pathologically characterized by progressive structural and functional deterioration of intervertebral discs (Dowdell et al., 2017). Although surgical and pharmacological interventions have achieved partial advancements in recent years, IVDD remains highly prevalent with limited therapeutic efficacy for reversal or complete resolution (Smith et al., 2011; Wu et al., 2020). Consequently, deciphering the molecular underpinnings of disc degeneration and developing superior treatment modalities represent urgent priorities. Substantial evidence indicates that IVDD pathogenesis involves dysregulated cellular processes including aberrant cell death, oxidative stress, inflammatory/immune dysregulation, and extracellular matrix (ECM) metabolic imbalance (He et al., 2023a; Xia et al., 2024). Emerging studies now identify copper homeostasis as a critical modulator in IVDD progression (He et al., 2023b; Staszkiewicz et al., 2023). Dysfunctional copper metabolism elevates free copper ion concentrations within the disc microenvironment, which exacerbates oxidative stress via Fenton reactions, directly inflicts cellular damage, and disrupts ECM homeostasis (Chen H. et al., 2024) Furthermore, copper ions act as co-activators of inflammatory signaling pathways (NF-κB), upregulating matrix metalloproteinases (MMPs) and pro-inflammatory cytokines such as IL-1β, TNF-α, thereby establishing a self-amplifying feedback loop of “copper accumulation → oxidative damage → inflammatory amplification → ECM degradation” (Chen J. et al., 2024). Additionally, mitochondria-dependent cuproptosis accelerates IVDD by promoting nucleus pulposus cell apoptosis through the FDX1-mediated protein lipoylation axis. In a recent study, Chen X. et al. (2024) investigated cuproptosis in IDD using *in vitro* and *in vivo* models, elucidating the interplay between oxidative stress and copper sensitivity in nucleus pulposus cells (NPCs). Their findings revealed that oxidative stress activates the SP1-CTR1 axis, increasing intracellular copper influx and synergistically upregulating FDX1 expression. This drives abnormal aggregation of TCA cycle-related proteins and cuproptosis, underscoring the central role of cuproptosis in IDD progression and proposing the SP1/FDX1 pathway as a novel therapeutic target. Zhang C. et al. (2023) identified eight cuproptosis-related prognostic regulators and two molecular subtypes, constructing a nomogram model to accurately predict IDD risk, thereby offering potential biomarkers and immunotherapeutic strategies for IDD. Additionally, a recent biomarker study on intervertebral disc degeneration (IVDD) revealed significant upregulation of miR-15a-5p in degenerative discs, demonstrating its multifaceted regulatory roles in core IVDD pathomechanisms. Bioinformatic analyses identified its potential targets across multiple IVDD-associated pathways, including copper-related genes (CRGs), ferroptosis regulators, oxidative stress mediators, and immune-related factors. These findings highlight the miR-15a-5p-mRNA regulatory axis as a promising therapeutic target for precision intervention in IVDD (Li C. et al., 2023).

### 5.5 Osteosarcoma and cuproptosis

Osteosarcoma (OS), a malignant mesenchymal tumor predominantly affecting children and adolescents, is often diagnosed at advanced stages with distant metastases due to asymptomatic early progression (Biazzo and De Paolis, 2016). Long-term survival rates remain suboptimal (Wittig et al., 2002). Limited therapeutic advances over the past three decades, particularly for multidrug-resistant, relapsed, or metastatic patients, underscore the urgent need for prognostic biomarkers and novel therapeutic targets.

Current research on cuproptosis in OS focuses on CRGs and RNA fragments (Bian et al., 2023; Feng et al., 2023; Gao et al., 2025; Hu et al., 2023; Huang et al., 2023; Jiang et al., 2022; Jiang M. et al., 2023; Liu C. et al., 2022; Ni et al., 2023; Wang et al., 2023c; Yang et al., 2022). Dysregulated CRGs effectively predict OS diagnosis, prognosis, tumor immune microenvironment (TME), and immunotherapy response (Han et al., 2024). Yang et al. (2022) established a prognostic model using four CRGs (LIAS, LIPT1, BCL2L1, PDK1), demonstrating strong associations between CRG signatures and TME, positioning CRGs as reliable prognostic indicators for OS. Jiang J. et al. (2023) identified PDHA1 and CDKN2A as optimal diagnostic CRGs for OS. Survival analysis of 10 CRGs revealed that high CDKN2A and FDX1 expression correlates with poorer survival, while elevated LIAS, LIPT1, and PDHA1 predicts better outcomes. Hu et al. (2023) found that the high levels of FDX1 in patients with poor prognosis for OS show a strong correlation, suggesting that it may serve as a promising prognostic indicator. Similarly, another study demonstrated that elevated expression of FDX1 promotes osteosarcoma (OS) cell migration, thereby exacerbating tumor malignancy Feng et al. (2023). Consequently, targeted inhibition of FDX1 represents a promising therapeutic strategy for OS. Moreover, long non-coding RNAs (lncRNAs) as key regulators of tumor pathogenesis, invasion, and progression (Bian et al., 2023). Cuproptosis-related lncRNAs (CRLs) may serve as prognostic biomarkers, potentially modulating OS aggressiveness and outcomes via copper metabolism and cell death pathways (Gao et al., 2025; Huang et al., 2023; Jiang et al., 2022; Liu P. et al., 2022; Ni et al., 2023; Wang et al., 2023d; Xie et al., 2023). CRLs are linked to cancer-associated fibroblasts (CAFs) in OS, offering insights for survival prediction and therapy. Targeting lncRNAs involved in copper metabolism—through small molecules, siRNA, or antisense oligonucleotides (ASOs)—may control cuproptosis and tumor progression. Multi-algorithm analyses of TME and immune status across risk groups reveal higher stromal scores and CAF infiltration in low-risk patients, contrasting with upregulated immune cell subsets in high-risk cohorts, providing a framework for clinical decision-making and personalized therapies (Wang A. et al., 2023; Xie et al., 2023).

In terms of treatment strategies, copper alloys exhibit exceptional hemocompatibility, antibacterial activity, and osteogenic potential during post-resection recovery (Elborolosy et al., 2023). Cu^2+^enhance chemodynamic therapy (CDT) efficacy by depleting glutathione (GSH) via redox reactions (Ye et al., 2024). Copper complexes (25–100 μM) selectively inhibit OS cell viability, with significantly higher toxicity toward tumor osteoblasts than normal cells (Leon et al., 2014). Other copper-based agents similarly demonstrate OS-specific cytotoxicity with minimal osteoblast damage (Balsa et al., 2023). Chemotherapy remains indispensable in OS treatment, with cisplatin, paclitaxel, and etoposide showing distinct IC50 values between risk groups (Huang et al., 2024). Cuproptosis signatures may predict immunotherapy response and chemosensitivity. Copper chelators can also play a certain role in tumor therapy, using targeted selective action on cancer cells, inducing apoptosis of cancer cells through oxidative stress and other mechanisms (Ismail et al., 2022). At the same time, copper chelating agent can also inhibit the proliferation of vascular endothelial cells through the interaction mechanism with copper, thus playing a role in inhibiting tumor growth (Gupte and Mumper, 2009).

Targeted therapies leveraging the high intrinsic copper content of OS cells are under exploration. Copper metallocompounds may exacerbate intracellular copper overload to induce cytotoxicity, offering novel antitumor avenues. Promising agents include milciclib malate, HMN-214, GSK461364, abemaciclib, palbociclib, and PF-477736, validated as potential OS-targeted drugs (Han et al., 2024).

### 5.6 Rheumatoid arthritis and cuproptosis

Rheumatoid arthritis (RA), an autoimmune disease, primarily manifests as symmetric, progressive polyarthritis affecting multiple small joints (Turk et al., 2023). Persistent synovial inflammation leads to cartilage, bone, and periarticular soft tissue damage, resulting in joint deformities and functional impairment (Radu and Bungau, 2021; Smolen et al., 2016). Elevated serum copper levels in active RA patients correlate positively with erythrocyte sedimentation rate (ESR) and morning stiffness, and inversely with hemoglobin levels, suggesting serum copper as a biomarker for RA disease activity (Xin et al., 2015). During RA progression, cuproptosis exacerbates chronic inflammation by catalyzing ROS production via the tricarboxylic acid (TCA) cycle, inducing oxidative stress that damages vasculature and connective tissues (Smolen et al., 2016). copper-related genes further regulate immune cell metabolism, driving pro-inflammatory cytokine release, autoantigen-antibody reactions, and immune complex deposition, perpetuating joint destruction (Xu et al., 2025).

Given excessive copper levels in RA, Zhao et al. (2022) demonstrated that copper induces cell death by binding to lipoylated TCA cycle components, promoting lipoylated protein aggregation and proteotoxic stress. This mechanism may affect diverse RA-associated cells, including fibroblast-like synoviocytes, effector T cells, and macrophages, contributing to inflammation, pannus formation, and bone erosion. RA patients exhibit a twofold higher incidence of osteoporosis compared to healthy populations, with studies linking copper dysregulation to osteoporotic development (Liu Y. et al., 2022). Hypoxic bone environments and glycolytic energy metabolism suppress cuproptosis, potentially enhancing survival and proliferation of osteoblasts, osteoclasts, effector T cells, and macrophages, thereby mediating osteoporosis (Tsvetkov et al., 2022). Zhou et al. (2023) integrated bioinformatics and experimental validation to conclude that cuproptosis-related genes regulate RA by modulating inflammatory factor secretion and macrophage metabolism. Ten cuproptosis-associated genes are implicated in RA processes: PDHA1 regulates glycolysis and inflammation; microRNAs (miRNAs) primarily target PDHB; GLS1 and LIPT1 modulate glutamine metabolism; DLAT governs mitochondrial function and TCA cycle activity; FDX1influences fatty acid oxidation and steroidogenesis; MTF1 and LIAS regulate copper homeostasis; HIF-1 and CDKN2A mediate cellular senescence (Zhao et al., 2022). Wang et al. found seven CRGs (ATP7A, FDX1, LIAS, LIPT1, DLD, MTF1, CDKN2A) were significantly differentially expressed in RA patients, and six of them (except MTF1) were upregulated in RA (Wang et al., 2023c). Jiang et al. also found high expression of CRGS such as DLST, DLD, and ATP7A in RA (Jiang X. et al., 2023). These genes can be used as new biomarkers of RA, and targeting the cuproptosis pathway or regulating immune infiltration may become a new strategy for RA treatment.

Additionally, copper promotes ferroptosis by inducing autophagic degradation of glutathione peroxidase 4 (GPX4) (Xue et al., 2023). Downregulation of the GSH-GPX4 axis, cystine/glutamate antiporter, and nuclear factor erythroid 2-related factor 2 (Nrf2) triggers synovial cell ferroptosis, exacerbating RA synovitis (Datta et al., 2014).

### 5.7 Spinal cord injury and cuproptosis

Spinal cord injury (SCI), a severe central nervous system trauma, causes permanent loss of motor, sensory, and autonomic functions below the injury level, with limited recovery (Anjum et al., 2020). Its pathology involves primary injury and progressive secondary injury cascades. Secondary injury impairs mitochondrial homeostasis, triggering calcium overload, excitotoxicity, and oxidative stress, which exacerbate neuronal damage (McDonald and Sadowsky, 2002). Mitochondrial dysfunction observed in these processes accelerates neuronal death and inhibits regeneration (Fan et al., 2018) Programmed cell death (PCD) secondary to SCI—including apoptosis, necroptosis, pyroptosis, ferroptosis, cuproptosis, and autophagy—is a critical barrier to functional recovery (Song et al., 2024). While physiological PCD may serve protective roles, excessive PCD exacerbates SCI by damaging surrounding neural tissue. Gene expression analyses reveal stage-specific PCD patterns post-SCI, suggesting therapeutic strategies to inhibit PCD pathways and induce autophagy. Interventions targeting hub genes associated with these pathways may also offer therapeutic potential (He et al., 2023a).

Dihydrolipoamide dehydrogenase (DLD), a regulator of copper toxicity, is significantly upregulated after acute SCI (ASCI) and correlates with disease severity (Li K. et al., 2023). DLD exacerbates ASCI by promoting copper toxicity, which disrupts the immune microenvironment, enhances polarization of peripheral M2 macrophages, and induces systemic immunosuppression. Post-ASCI, DLD facilitates copper binding to lipid components of the TCA cycle in peripheral blood, driving cuproptosis. This cascade disrupts immune homeostasis, alters macrophage polarization, aggravates SCI-induced immunosuppression syndrome (SCI-IDS), and worsens ASCI outcomes (Liu et al., 2022). Further analyses confirm elevated M2 macrophage infiltration in high-grade ASCI patients, correlating positively with ASIA impairment scale scores and DLD expression. Thus, DLD overexpression post-ASCI may drive adverse prognosis via macrophage polarization, positioning DLD as a therapeutic target (Li Y. et al., 2023). Mao et al. identified Mpeg1 as a hub gene related to cuproptosis, which may alleviate spinal cord tissue injury by regulating the infiltration of immune cells, such as M2 macrophages, and inhibiting inflammatory responses (Mao et al., 2025).

Mitochondrial dysfunction post-SCI amplifies injury cascades, making cuproptosis inhibition a potential therapeutic strategy. Preserving mitochondrial integrity and mitigating copper-induced damage could enhance neuronal survival and functional recovery.

### 5.8 Osteomyelitis and cuproptosis

Osteomyelitis, a common orthopedic infection, is typically caused by bacteria, *Pseudomonas aeruginosa*, or fungi, with *Staphylococcus aureus* (*S. aureus*) being the most prevalent pathogen (Lew and Waldvogel, 2004). Current diagnostic challenges include the nonspecificity of inflammatory biomarkers and the limited sensitivity of magnetic resonance imaging (MRI) in early-stage disease (Lew and Waldvogel, 1997). Thus, developing effective early diagnostic methods remains an urgent priority.

The pathogenesis and therapeutic strategies for osteomyelitis involve immune responses, with cuproptosis potentially playing a role. *S. aureus* biofilms evade host receptor recognition, largely mediated by Staphylococcal Protein A (SPA). SPA interacts with osteoclasts and osteoblasts, driving inflammatory cascades (Claro et al., 2011; Mendoza Bertelli et al., 2016). *S. aureus* also induces osteoblast death and bone destruction. Mendelsohn et al. (2023) observed mitochondrial dysfunction in chronic osteomyelitis patients, including ROS accumulation characteristic of cuproptosis. Furthermore, inhibitors targeting the programmed cell death protein 1/programmed death-ligand 1 (PD-1/PD-L1) pathway reduce mitochondrial autophagy in macrophages during S. aureus-induced osteomyelitis, thereby attenuating inflammation (Li C. et al., 2023). Inducing cuproptosis may represent a novel antibiotic-free therapeutic approach for methicillin-resistant *S. aureus* (MRSA)-associated osteomyelitis. Shi et al. (2024) showed that comparative analysis of CRGs and immune microenvironments between S. aureus-infected osteomyelitis patients and healthy controls identified three signature M2R-CRGs: SLC31A1, DLD, and MTF1. Among these, SLC31A1 likely modulates the immunomicroenvironment by regulating M2 macrophage polarization, MTF1 exerts anti-inflammatory effects via M2 macrophage activation, and DLD promotes inflammatory responses and accelerates osteoblast death by suppressing M2 macrophage activity (Duarte et al., 2021). A diagnostic model based on these CRGs enables early prediction of osteomyelitis risk.

Therapeutically, targeted delivery of copper ions to infection sites to trigger bacterial cuproptosis represents a promising strategy. Cu^+^ enhances the efficiency of iron-based Fenton reactions by facilitating Fe^3+^ reduction to Fe^2+^ (Koo et al., 2022). Concurrently, Cu^+^ catalyzes the generation of highly toxic hydroxyl radicals (·OH) through Fenton-like reactions (Ma et al., 2023). Li et al. (2025) developed a bone marrow mesenchymal stem cell (BMSC) membrane-engineered nanovesicle (CFE@CM) with dual bone-targeting and cuproptosis-inducing capabilities. Upon reaching osteomyelitic lesions, CFE@CM disassembles, releasing free Cu^+^, Fe^2+^, ELC, and H_2_O_2_, synergistically activating Fenton reactions and cuproptosis mechanisms. Qiu et al. have developed a nanoheterojunction catalytic reactor consisting of copper ferrite (CuFe2O4) and molybdenum disulfide (MoS2) quantum dots (CFO@MoS2) to induce the cuproptosis of bacteria using ultrasonically catalyzed binding of copper ions (Qiu et al., 2025) (Table 3).

**TABLE 3:** Key findings on association of Cuproptosis with musculoskeletal diseases.

Diseases	Study	Evidence type	Key findings
Osteoarthritis	Zhou et al. (2021)	Bioinformatic prediction	In OA patients, the expressions of FDX1 and LIPT1 were upregulated, while the expressions of DBT and DLST were downregulated
	He et al. (2024)	Bioinformatic prediction *In vitro*	CDKN1A, FZD7, GABARAPL2, and SLC39A14 are excellent biomarkers and potential therapeutic targets for OA.
	Li et al. (2024)	*In vitro* *In vivo*	1. Cuprorivaite microspheres significantly improved IL-1β-induced chondrocyte injury and cartilage tissue injury in OA mouse models by inhibiting inflammation, oxidative stress, and cuproptosis 2. The mechanism of action may involve inhibition of the Wnt/β-catenin pathway
Osteoporosis	Chen et al. (2024d)	Bioinformatic prediction *In vivo*	1. Cuproptosis plays an important role in the pathogenesis of osteoporosis and is closely related to the immune microenvironment 2. The key genes identified (MAP2K2, FDX1, and COX19) may serve as potential biomarkers for the diagnosis and treatment of osteoporosis
	Liu et al. (2024)	*In vitro*	1. Mitochondrial dysfunction plays an important role in the pathogenesis of osteoporosis 2. Cuproptosis can cause mitochondrial dysfunction, which affects the occurrence and progression of osteoporosis
	Wang et al. (2024c)	*In vitro* *In vivo*	CUBA particles have good biocompatibility and osteogenesis ability *in vitro*, and have shown significant therapeutic effects in a mouse model of osteoporosis, which can increase bone mineral density and bone microstructure parameters, inhibit osteoclast formation, and promote new bone formation
Sarcopenia	Chen et al. (2021)	Bioinformatic prediction *In vitro*	1. High dose copper induced apoptosis by inducing nucleolar stress and interfering with ribosome synthesis pathway 2. In Cu treated cells, nucleolar morphology was changed, rRNA processing was blocked, protein synthesis was inhibited, and mitochondrial function was impaired
	Zhu et al. (2023)	Bioinformatic prediction	1. Four key genes for cuproptosis associated with sarcopenia (PDHA1, DLAT, PDHB, and NDUFC1) were identified and a diagnostic model with high predictive value was constructed 2. These genes play important roles in energy metabolism and mitochondrial function and may serve as diagnostic biomarkers for sarcopenia
Intervertebral Disc Degeneration	Chen et al. (2024a)	*In vivo* *Invitro*	1. The expression of cuproptosis related genes was increased in the degenerated intervertebral disc 2. The expression of CTR1 and ATP7A increased under oxidative stress. The expression of SP1 increased under oxidative stress 3. SP1 inhibition mitigated disc degeneration in rat IDD models
	Zhang et al. (2023b)	*In vivo* *Invitro* Bioinformatic prediction	1. The expression level of cuproptosis gene in IDD was verified by *in vitro*and *in vivo*experiments 2. The results showed that the expression levels of FDX1, LIAS, LIPT1, GCSH, DLST, DLAT and PDHB in IDD samples were significantly decreased, while the expression levels of ATP7A, ATP7B and MTF1 were significantly increased
Osteosarcoma	Bian et al. (2023)	*In vitro* Bioinformatic prediction	1. Copper ions promote cancer cell proliferation, angiogenesis and metastasis by activating various signaling pathways (such as RTK, PI3K-AKT, MAPK, etc.) 2. In a variety of tumors, the expression level of CRGs is closely related to the prognosis of patients 3. In hepatocellular carcinoma, high expression of CDKN2A is associated with a worse prognosis
	Hu et al. (2023)	*In vitro* Bioinformatic prediction	1. There are two different expression patterns of cuproptosis genes in osteosarcoma patients, and high expression of FDX1 is associated with poor prognosis in osteosarcoma patients 2. PLCD3 promotes proliferation and migration in osteosarcoma cells
	Jiang et al. (2022)	Bioinformatic prediction	The study identified 431 lncrnas associated with cuproptosis, of which 109 were downregulated and 185 upregulated in osteosarcoma
	Jiang et al. (2023a)	Bioinformatic prediction	Through artificial intelligence technology, PDHA1 and CDKN2A were successfully identified as cuproptosis-related biomarkers of osteosarcoma, and their potential applications in diagnosis and immunotherapy were validated
	Yang et al. (2022)	*In vitro* Bioinformatic prediction	1. Six differentially expressed CRGs associated with osteosarcoma were identified 2. These genes were highly expressed in osteosarcoma and significantly enriched in the citric acid cycle (TCA cycle), pyruvate metabolism, glycolysis/gluconeogenesis, carbon metabolism and other pathways
	Gao et al. (2025)	Bioinformatic prediction	1. A total of 4,811 differentially expressed genes were identified, among which PDHA1 and CDKN2A were significantly differentially expressed between osteosarcoma and controls 2. PDHA1 and CDKN2A were identified as specific cuproptosis-related biomarkers for osteosarcoma
	Wang et al. (2023a)	*In vitro* Bioinformatic prediction	1. The expression level of ZNF37BP in osteosarcoma cell lines was significantly higher than that in normal osteoblasts, while the expression levels of ATP7A, LIPT1, AL353759.1 and AC005034.5 in osteosarcoma cell lines were significantly lower than that in normal osteoblasts
	Lin et al. (2025)	*In vitro* *In vivo*	1.CYFIP1 overexpression upregulated the expression of AURKAIP1 and FDX1, resulting in mitochondrial translation dysregulation and elevated ROS levels, which ultimately triggered cuproptosis of OS cells and inhibited tumor growth
Rheumatoid Arthritis	Xu et al. (2025)	Bioinformatic prediction *In vitro* *In vivo*	1. Eleven cuproptosis related genes associated with RA were identified, including DLST, LIAS, DLAT, DLD, PDHB, LIPT1, DBT, ATP7B, SLC31A1, FDX1, and PDHA1 2. PDHB protein levels were significantly reduced in CIA model rats 3. PDHB may play an important role in RA development
	Liu et al. (2022a)	*In vitro*	1. Copper ions regulate the immune response by participating in the signal transduction and metabolic processes of immune cells 2. Copper ions can promote the proliferation and osteogenic differentiation of mesenchymal stem cells and enhance their role in bone tissue repair 3. Copper ions can also affect the role of mesenchymal stem cells in inflammation and immune response by regulating their immunomodulatory function
	Zhou et al. (2023)	Bioinformatic prediction *In vivo*	1. In RA patients, 7 out of 13 CRGS showed significantly increased expression levels, while DLST expression levels were significantly decreased 2. These differentially expressed CRGS are strongly implicated in the pathogenesis of RA. 3. The important role of these genes in RA was further supported by the significantly increased expression levels of five predicted genes (FAM96A, MAK4P3, PRPF39, SLC35A1, TMX1) validated by qRT-PCR in the animal model of RA.
Spinal Cord Injury	Li et al. (2023a)	Bioinformatic prediction *In vitro*	1. The expression of DLD genes is significantly upregulated in patients with ASCI and is significantly related to the occurrence of ASCI. 2. The significant increase in mRNA and protein levels of DLD in peripheral blood leukocytes of ASCI patients further supports the important role of DLD in ASCI.
	Mao et al. (2025)	Bioinformatic prediction	1. As a key gene related to cuproptosis, Mpeg1 may alleviate spinal cord injury by regulating the infiltration of immune cells (such as M2 macrophages) and inhibiting inflammatory response 2. Targeting the Mpeg1 and cuproptosis pathways provides a new strategy for SCI treatment
Osteomyelitis	Li et al. (2023b)	*In vitro* *In vivo*	1. PD-1/PD-L1 signaling inhibits the bactericidal activity of macrophages by activating mitophagy in *S. aureus* osteomyelitis, leading to bone destruction 2. Blocking PD-1/PD-L1 signaling can significantly enhance the bactericidal ability of macrophages and reduce bone destruction, which provides a new strategy for the treatment of *S. aureus* osteomyelitis
	Shi et al. (2024)	Bioinformatic prediction *In vivo*	1. In osteomyelitis samples, SLC31A1, DLD, and MTF1 expression was significantly upregulated, whereas GLS and DBT expression was significantly downregulated 2. In the rat model of *Staphylococcus aureus* induced osteomyelitis, the mRNA and protein expression levels of SLC31A1, DLD and MTF1 were significantly upregulated, which was consistent with the bioinformatics results

## 6 Therapeutic strategies based on copper homeostasis and CRGS

According to the description of the association between the above diseases and cuproptosis, treatment methods include the use of nanomaterials or biomaterials to deliver copper ions to the corresponding areas for treatment, as well as to combat ROS and oxidative stress generated by cuproptosis. In addition to those, there are two other different therapeutic strategies for musculoskeletal diseases by cuproptosis, which include two aspects: the regulation of copper homeostasis by copper chelators and copper ionophoresis, and the gene and epigenetic regulation of CRGs.

### 6.1 Regulating copper homeostasis

Copper chelators reduce the adverse effects of copper on cells mainly by binding free copper ions in the body and promoting their excretion, while copper ionophores regulate the death of cells mainly by affecting the intake and expulsion of copper ions (Zhao et al., 2022).

The use of copper chelators may be an acceptable treatment for rheumatoid arthritis, and high copper levels in RA are an important factor in the development of the condition (Xu et al., 2025). At present, D-penicillamine and ethylenediamine tetraacetic acid have been used in the treatment of RA, and certain results have been achieved (Bamonti et al., 2011; Kumar et al., 2021), But there are still some side effects (Kumar et al., 2021). Therefore, in the future, how to better use copper chelating agent to regulate the copper level in patients with bone and joint diseases and minimize the occurrence of side effects is a possible research direction. Copper chelators can also play a certain role in tumor therapy, using targeted selective action on cancer cells, inducing apoptosis of cancer cells through oxidative stress and other mechanisms (Ismail et al., 2022). At the same time, copper chelating agent can also inhibit the proliferation of vascular endothelial cells through the interaction mechanism with copper, thus playing a role in inhibiting tumor growth (Gupte and Mumper, 2009). Conventional cancer therapies such as chemotherapy and radiotherapy suffer from limitations including poor targeting specificity and significant adverse effects, resulting in suboptimal therapeutic outcomes (Yang W. M. et al., 2023). Although immunotherapy has emerged as an advanced approach in recent years, it still faces challenges of narrow anticancer spectrum and complex treatment-related complications (Peng et al., 2022; Winer et al., 2018). Currently, therapeutic strategies targeting cuproptosis mechanisms show particular promise for osteosarcoma treatment. Cui et al. developed copper-depleting nanoparticles that induce intracellular copper depletion and subsequent apoptosis in cancer cells (Li Y. et al., 2022).

Copper ionophore can increase the intracellular copper ion level and exert anticancer activity. Ionophore exerts toxic effects on cancer cells mainly by increasing ROS production and inhibiting proteasome (Denoyer et al., 2016; Xiao et al., 2010). Wu et al. designed an ES-Cu compound capable of releasing substantial copper ions within tumor cells to trigger cuproptosis (Wu J. et al., 2024). Compared to conventional approaches, these novel therapies demonstrate multiple advantages. For instance, chemotherapeutic agents utilizing cuproptosis mechanisms exhibit enhanced chemotherapy sensitivity (Wen et al., 2023), while combination therapies with emerging modalities like phototherapy and sonotherapy achieve superior targeting precision and deeper tissue penetration (Chen et al., 2023; Ning et al., 2023).

### 6.2 Gene and epigenetic regulation

Editing and regulating copper-death related genes is also a method with potential application value to use the copper-death mechanism to treat musculoskeletal diseases. It can enhance mitochondrial respiration and promote cuproptosis of abnormal proliferation cells by activating positive regulatory genes (FDX1, PDHA1). Inhibition of negatively regulated genes (MTF1, GLS) blocked hypoxic adaptation and glycolytic resistance to cuproptosis. For example, the use of histone deacetylase inhibitors (such as FK228) in the treatment of RA can upregulate CDKN2A and induce FLS senescence and apoptosis. Use of GLS inhibitors such as CB-839 to reduce Th17 differentiation and FLS proliferation (Zhao et al., 2022). Epigenetic regulation includes DNA methylation, histone modification (Knott and Doudna, 2018). The pharmacological management of osteoporosis primarily involves bone resorption inhibitors (e.g., bisphosphonates), bone formation promoters (e.g., parathyroid hormone), and bone metabolism regulators (e.g., calcium supplements). Current research indicates that miRNAs encapsulated in extracellular vesicles (EVs) may influence osteoblast function through modulation of cuproptosis-related genes. For instance, miR-21-5p has been demonstrated to target PDHA1, thereby suppressing mitochondrial oxidative phosphorylation (Zhuang et al., 2021) Additionally, oxidative stress has been shown to upregulate miR-183-5p levels, which subsequently inhibits mesenchymal stem cell proliferation and induces cellular senescence (Davis et al., 2017). Emerging evidence suggests that targeted delivery of specific miRNAs via EVs to rebalance bone metabolism represents a promising therapeutic strategy for osteoporosis treatment (Sun et al., 2024). Lin et al. (2025) demonstrated that by regulating CYFIP1 to bind RNMT and promoting m7G methylation of target mRNA, abnormal AURKAIP1 leads to abnormal mitochondrial translation, and upregulation of FDX1 triggers cuproptosis. It provides a new target for the treatment of osteosarcoma.

## 7 Conclusions and perspectives

Numerous musculoskeletal disorders are closely associated with copper homeostasis and copper-induced cell death. The copper oxidation process primarily disrupts TCA cycle and may play a regulatory role in the progression of various bone and joint diseases. Cuproptosis exhibits dual attributes of being both a “core driving factor” and a “secondary outcome” in musculoskeletal diseases, depending on the disease type, pathological stage, and microenvironment context. In diseases such as osteoarthritis (OA) and osteosarcoma (OS), dysregulation of copper metabolism directly triggers cell death and inflammation via cuproptosis, serving as a key link in the pathological mechanism. In diseases like spinal cord injury (SCI) and osteomyelitis, cuproptosis is more of a manifestation of metabolic disorders following tissue damage and is regulated by other pathological processes (such as hypoxia and infection). It is necessary to further distinguish the causal timing of cuproptosis, combine multi-omics analysis and clinical intervention trials, and clarify its dominant position in specific diseases to guide the development of precision treatment strategies. Currently, many researchers are focusing on investigating the relationship between copper-induced cell death and major pathological conditions. Although certain clinical treatments for bone and joint diseases exist, most remain insufficiently effective. Targeting cuproptosis provides novel avenues for developing therapeutic strategies. Through systematic investigation of common musculoskeletal disorders and the correlation analysis between disease manifestations and specific genes involved in cuproptosis, it can be inferred that these genes likely play critical roles in the pathogenesis of musculoskeletal diseases. The discovery of cuproptosis has also deepened our understanding of these disorders and their underlying molecular mechanisms. Furthermore, cuproptosis holds potential value for screening therapeutic agents targeting these diseases.

Future research directions may emphasize strategies leveraging the strong chelating properties of copper chelators to reduce intracellular copper levels or inhibit copper transporters, thereby suppressing cuproptosis—an approach that offers innovative possibilities for disease intervention and treatment. Conversely, utilizing copper ionophores to enhance intracellular copper accumulation may exhibit therapeutic potential for osteosarcoma management. However, in-depth investigations into copper oxidases and their associated genes require extensive experimental exploration and present significant challenges. Despite these obstacles, this field remains highly promising with substantial potential for groundbreaking advancements in future research.
